# Extensively drug-resistant *Haemophilus influenzae* isolated in Geneva, Switzerland

**DOI:** 10.1007/s10096-025-05093-w

**Published:** 2025-03-06

**Authors:** Abdessalam Cherkaoui, Patrice Francois, Nadia Gaia, Gesuele Renzi, Adrien Fischer, Jacques Schrenzel

**Affiliations:** 1 Bacteriology Laboratory, Division of Laboratory Medicine, Department of Diagnostics, Geneva University Hospitals, 4 Rue Gabrielle-Perret-Gentil, 1205 Geneva, Switzerland; 2 Genomic Research Laboratory, Department of Molecular Microbiology, Faculty of Medicine, Geneva, Switzerland

**Keywords:** *Haemophilus influenzae*, Extensively drug resistant, Ceftriaxone resistance, Penicillin-binding protein 3 amino acid substitutions

## Abstract

The emergence of multi-drug resistant (MDR) and even extensively drug-resistant (XDR) strains among *H. influenzae* was observed in some Asian countries. Herein, we reported the first XDR *H. influenzae* isolated in Geneva, Switzerland. This strain was isolated in a good-quality sputum sample from a 63 year-old male patient. There was no respiratory infection diagnosed at that time. The strain was non-typeable and pan-β-lactam resistant. According to whole genome sequencing analysis it belongs to sequence type 159 and the ST-107 clonal complex. It was classified into group III + regarding the amino acid substitutions identified in the transpeptidase domain of PBP3.

Nowadays *H. influenzae* remains an important pathogen. Non-typeable strains have become the most frequently recovered strains in both invasive and non-invasive diseases [1]. Due to the development of several drug-resistance mechanisms, penicillins as well as first and second generation cephalosporins are increasingly becoming less effective against *H. influenzae* [2]*.* In addition, recent reports have highlighted the emergence of multi-drug resistant (MDR) and even extensively drug-resistant (XDR) strains among *H. influenzae* [2–5]*.* The first MDR *H. influenzae* strain was reported in West Germany in 1980. Since then, *H. influenzae* strains resistant to at least one agent in three or more classes of antibiotics were reported in different countries [6–8]. According to Magiorakos et al*.* a bacterial isolate is classified as XDR when it remains susceptible to antimicrobial drugs from at most two classes of antibiotic drugs [9].

Herein, we reported the first XDR *H. influenzae* isolated in Geneva, Switzerland. This *H. influenzae* strain was isolated in a good-quality sputum sample from a 63 year-old male patient. It was non-typeable. There was no respiratory infection diagnosed at that time. This highly resistant strain was considered as selected by a previous antimicrobial treatment (co-amoxicillin).

The identification was performed by matrix-assisted laser desorption ionization–time of flight mass spectrometry (MALDI-TOF MS) (Bruker Daltonics, Bremen, Germany).

The antimicrobial drug susceptibility profile was defined by disc diffusion according to the EUCAST guidelines. Briefly, a 0.5 McFarland standard, prepared by picking several colonies from overnight growth on chocolate agar, was spread over the entire surface of the Mueller–Hinton agar + 5% defibrinated horse blood and 20 mg/L β-NAD (MH-F) (bioMérieux). The antibiotic disks were then dispensed, and the MH-F plates incubated in 5% CO_2_ atmosphere at 35 ± 1ºC during 18 ± 2 h.

Etest® strips (bioMérieux) were used to determine the minimum inhibitory concentrations (MICs) of the antimicrobial agents according to the manufacturer's instructions. Interpretation of the MICs and the inhibition zone diameters for the drugs included in this study was performed using Eucast breakpoint tables, version 15.0 (2025).

Using the disk diffusion method, this strain was reported resistant to all β-Lactam antibiotics tested, in addition to fluoroquinolones, cycline drugs, and co-trimoxazole. The strain remained only susceptible to rifampicin and chloramphenicol (Table 1). It is therefore classified as XDR. Regarding the β-lactams, the MICs were 256 mg/l for ampicillin, 6 mg/l for co-amoxicillin, 0.5 mg/l for piperacillin-tazobactam, 16 mg/l for cefuroxime, 0.25 mg/l for ceftriaxone, 1 mg/l for cefepime, and 8 mg/l for imipenem (Table 1). This strain was β-lactamase producing according to the cefinase assay. Hence, it was reported as a β-lactamase-positive co-amoxicillin-resistant strain (BLPACR). This result was confirmed by the presence of the gene *bla*_*TEM-1*_*.*

**Table 1:**
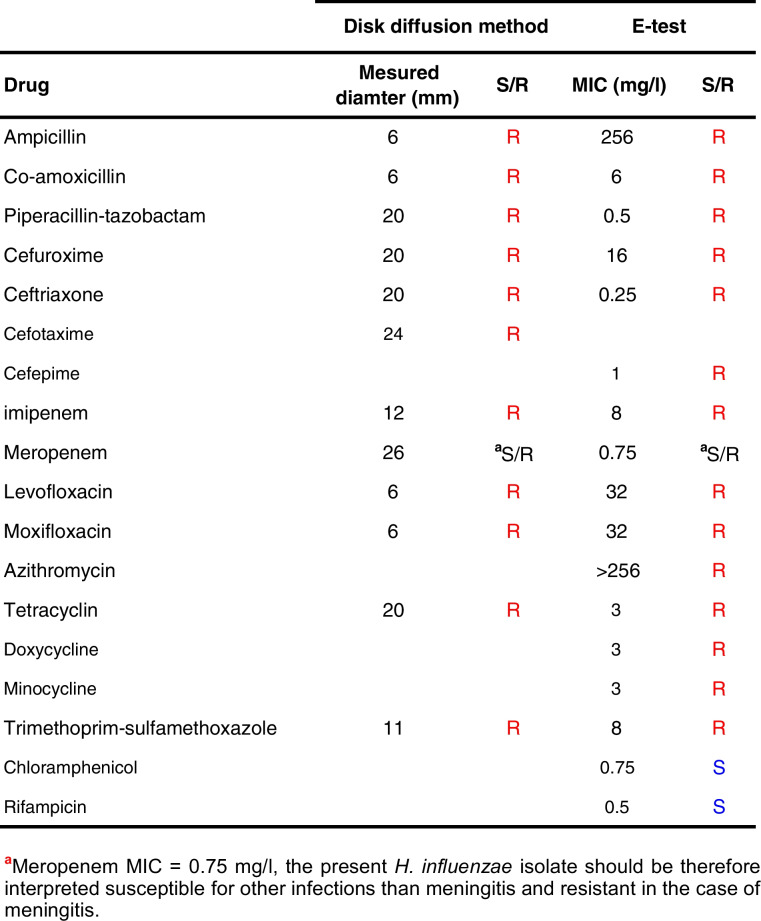
Susceptibility profile of the Geneva XDR *H. influenzae* isolate according to EUCAST breakpoint tables, version 15.0 (2025)

According to whole genome sequencing analysis it belongs to sequence type 159 and the ST-107 clonal complex. Sequence data that support the findings of this study have been deposited in the European Nucleotide Archive with the primary accession code PRJEB83694. DNA was purified using DNeasy columns (Qiagen). High-throughput sequencing was performed using the Illumina NovaSeq 6000 (Illumina, San Diego, California). Read quality was assessed with the Fastqc program (available at: http://www.bioinformatics.babraham.ac.uk/projects/fastqc/) and filtered using TRIMMOMATIC v0.39 (available at: http://www.usadellab.org/cms/?page=trimmomatic). Genome assembly was performed using Spades v3.15.5 with the following parameters: -k 21,33,55,77,99 –careful. Assembled genomes were annotated using the Prokka v1.10 program (PMID: 24,642,063). Specific resistance determinants within the genome sequence were assessed using resources available at the center for genomic epidemiology (https://www.genomicepidemiology.org/services/). The phylogenetic relationship of isolates was investigated by genomic single-nucleotide polymorphism (SNP)–based analysis using CSI Phylogeny (https://cge.food.dtu.dk/services/CSIPhylogeny/).

Regarding the resistance to β-lactam antibiotics, amino acid substitutions in the three conserved motifs of the active site of the transpeptidase domain of PBP3 were observed. Surrounding the KTG motif (Lys512-Thr-Gly) two amino acid substitutions were observed: Asn526Lys and Ala530Ser. Close to the SSN motif (Ser379-Ser-Asn), we identified the following three key substitutions: Met377Ile, Ser385Thr, and Leu389Phe. Finely, near to the STVK motif (Ser327-Thr-Val-Lys), the substitution Asp350Asn was identified (Table 2). This strain was classified into group III + according to PBP3 amino acid substitutions pattern observed [4].

**Table 2:** Amino acid substitutions observed in transpeptidase domain of *ftsI* gene

Group	Amino acid substitution for:																					
	Glu-	Ser-	Glu-	Ser-	Asp-	Ser-	Met-	Scer-	Leu-	Ala-	Ile-	Gly-	Ala-	Val-	Arg-	Asn-	Ala-	Thr-	Val-	Asp-	Ala-	Ala-
	141	273	274	311	350	357	377	385	389	437	449	490	502	511	517	526	530	532	547	569	586	587
III +	.	.	.	.	Asn	Asn	Ile	Thr	Phe	.	.	Glu	.	.		Lys	Ser	.	.	.	Ser	.

Several key mutations were identified in GyrA (Leu84Ser, Tyr88Asp, Asn201Lys, Ser353Ala, Ala407Ser, Val427Ala, Asp433Glu, and Glu740Asp); ParC (Thr60Ala, Ile84Ser, Lys206Gly, Asp276Glu, Ser478Asn, Lys587Glu, Ile675Met, and Val745Ile); ParE (Thr61Pro, Asp128Asn, Glu135Lys, Val136Ile, Val152Ile, Thr164Ile, Thr245Ala, Asn420Asp, Ser542Asn); and GyrB (Arg80Gly, Ala156Ser, Asn163Glu, Thr166Ala, Val400Ala, Ala573Thr, Ser601Asn, Lys610Gln, Val620Ile, Asp625Glu, and Thr721Ser). As reported previously, the following substitutions Leu84Ser and Tyr88Asp in GyrA; Ile84Ser in ParC; and Asn420Asp in ParE were associated with high-level resistance to fluoroquinolones [10].

The genome analysis indicated the presence of *msr*(D) and *mef*(A). These genes were carried by the transposon Tn6009-7 (accession #: EU399632). As highlighted previously, *msr*(D) shows similarities to *msr*(A) gene that encodes for an ATP transporter involved in the efflux transport of erythromycin and streptogramin B [11]. The sequence analysis did not reveal the presence of the Ala2058Gly mutation in the domain V of the 23S rRNA, which was observed in some persistent *H. influenzae* strains highly resistant to azithromycin [12]. No plasmid was observed in our strain.

To put the XDR Geneva strain in the international context, the resistant strains (accession numbers CP121103, CP121104, CP121105) and 2018-Y40 responsible for outbreaks in Japan (AP022867.1), as well as the reference strains RD KW20 (L42023.1); R2866 (CP002277), M19346 (NZ_CP031243) and *H. parainfluenzae* NCTC10665 (LR134481.1) were used to build a phylogenetic tree and a pairwise SNP table. Our strain appears quite distant from the other strains displaying a resistant phenotype, as shown in Fig. 1.

**Fig. 1:**
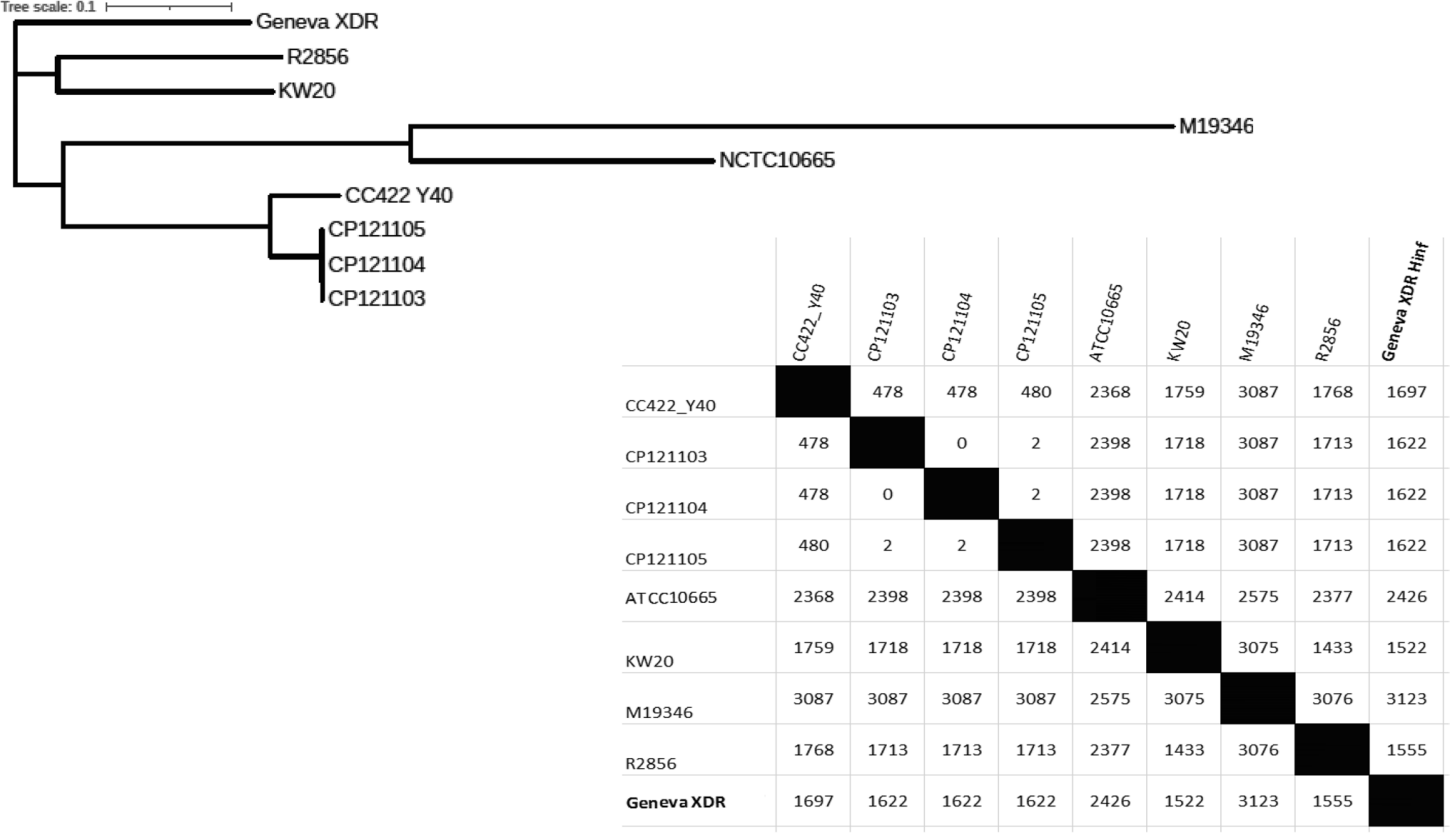
Phylogenetic tree and a pairwise SNP table

Over the past few years, significant changes in the antibiotic susceptibility patterns of *H. influenzae* were observed in several countries, with a tendency towards more resistant profiles. The resistance of *H. influenzae* to third-generation cephalosporins (3GC) is no longer considered as exceptional. In European countries, this resistance rate is estimated at 1 to 2% [13, 14]. Ser385Thr, and Leu389Phe substitutions in the transpeptidase domain of PBP3 in addition to other key mutations (i.e., Arg517His, and/or Asn526Lys) were noticed in the 3GC-resistant *H. influenzae* strains [4, 7, 15, 16]. Defining the distribution of these resistance determinants will help defining appropriate molecular diagnosis tools.

The present report provides a synthesis and analysis of the first XDR *H. influenzae* strain isolated in Geneva, Switzerland. The resistance to the β-lactams can be largely explained by the *ftsI* gene mutation pattern observed in this strain. Appropriate strategies are needed to hamper the development and spread of this high-level resistance in* H. influenzae.*

## Data Availability

Sequence data that support the findings of this study have been deposited in the European Nucleotide Archive with the primary accession code PRJEB83694.
